# Effect modification and non-collapsibility together may lead to conflicting treatment decisions: A review of marginal and conditional estimands and recommendations for decision-making

**DOI:** 10.1017/rsm.2025.2

**Published:** 2025-03-10

**Authors:** David M. Phillippo, Antonio Remiro-Azócar, Anna Heath, Gianluca Baio, Sofia Dias, A. E. Ades, Nicky J. Welton

**Affiliations:** 1 Bristol Medical School (Population Health Sciences), University of Bristol, Bristol, UK; 2 Methods and Outreach, Novo Nordisk Pharma, Madrid, Spain; 3 Child Health Evaluative Sciences, The Hospital for Sick Children, Toronto, ON, Canada; 4 Dalla Lana School of Public Health, University of Toronto, Toronto, ON, Canada; 5 Department of Statistical Science, University College London, London, UK; 6 Centre for Reviews and Dissemination, University of York, York, UK

**Keywords:** decision-making, effect modification, network meta-analysis, population adjustment

## Abstract

Effect modification occurs when a covariate alters the relative effectiveness of treatment compared to control. It is widely understood that, when effect modification is present, treatment recommendations may vary by population and by subgroups within the population. Population-adjustment methods are increasingly used to adjust for differences in effect modifiers between study populations and to produce population-adjusted estimates in a relevant target population for decision-making. It is also widely understood that marginal and conditional estimands for non-collapsible effect measures, such as odds ratios or hazard ratios, do not in general coincide even without effect modification. However, the consequences of both non-collapsibility and effect modification together are little-discussed in the literature.

In this article, we set out the definitions of conditional and marginal estimands, illustrate their properties when effect modification is present, and discuss the implications for decision-making. In particular, we show that effect modification can result in conflicting treatment rankings between conditional and marginal estimates. This is because conditional and marginal estimands correspond to different decision questions that are no longer aligned when effect modification is present. For time-to-event outcomes, the presence of covariates implies that marginal hazard ratios are time-varying, and effect modification can cause marginal hazard curves to cross. We conclude with practical recommendations for decision-making in the presence of effect modification, based on pragmatic comparisons of both conditional and marginal estimates in the decision target population. Currently, multilevel network meta-regression is the only population-adjustment method capable of producing both conditional and marginal estimates, in any decision target population.

## Highlights

1. **What is already known**
  When using non-collapsible measures of treatment effects, such as odds ratios or hazard ratios, marginal and conditional estimands have different interpretations and will not generally coincide, even in the absence of effect modification. The presence of effect modification means that there may not be a single most effective treatment for all individuals or subgroups in the population.
2. **What is new**
  We argue that population-average conditional and marginal estimands both quantify average effectiveness over a population but correspond to different decision questions, either to maximise the average effect for individuals in the population, or to minimise (or maximise) average event probabilities respectively. When effect modification is present, we show that these are no longer aligned and can result in conflicting treatment rankings. In such cases, making a single treatment decision can result in choosing an inferior treatment for the majority of individuals, or one with a worse expected number of events overall.
3. **Potential impact**
  We provide recommendations for decision-making in the presence of effect modification, for decisions based both purely on effectiveness and on cost-effectiveness. ML-NMR is at present the only population adjustment method that can produce the necessary estimates in any target population of interest. Where allowable, making decisions by subgroups may result in patients being given a more effective treatment for them and result in greater cost-effectiveness overall.

## 1 Introduction

Healthcare decision makers are frequently tasked with selecting the most effective treatment from a set of two or more possible candidate treatments, either purely in terms of treatment effectiveness or as a balance of cost-effectiveness. This requires reliable estimates of treatment effects, which are typically obtained from one or more randomised controlled trials (RCTs). When multiple trials are available, indirect comparison or network meta-analysis methods are widely used to synthesise all the evidence in one coherent analysis, even when no single trial compares all relevant treatments of interest.^1^ ^–^ ^4^ Effect modifiers are factors that alter the relative effectiveness of a treatment compared to control; for example, if a treatment is more effective for patients with more severe disease or with certain biomarkers. The presence of effect modification has strong implications for healthcare decision-making. First, meta-analyses, indirect comparisons, and network meta-analyses may be biased if differences in effect modifiers are not accounted for. Population-adjustment methods such as multilevel network meta-regression (ML-NMR),^5^ matching-adjusted indirect comparison (MAIC),^6^ and simulated treatment comparison (STC)^7^ ^–^ ^9^ aim to adjust for differences between study populations using available individual patient data (IPD) from one or more studies. These methods are primarily concerned with adjusting for patient characteristics that may be effect modifiers; study-level effect modifiers related to the design or context of the trials such as treatment administration or co-treatments are typically perfectly confounded at the study level and may require alternative adjustment methods. Second, treatment decisions may differ between populations or between subgroups within a population, and so estimates of treatment effects must be produced for the relevant decision target population (or subgroup thereof). Whilst ML-NMR can coherently synthesise networks of any size and produce estimates in any target population, MAIC and STC are limited to pairwise indirect comparisons between two studies and can only produce estimates relevant to the population of the aggregate study in the indirect comparison.

For population-level decision making, we are typically interested in population-average measures of treatment effects, although as we demonstrate here these may not be sufficient when there is effect modification. Care is needed to ensure that methods are combining compatible estimates and to appropriately interpret the results, particularly when the effect measure of interest is non-collapsible, such as odds ratios or hazard ratios. A summary effect measure is non-collapsible when the population-average marginal effects cannot be expressed as a weighted average of the individual- or subgroup-specific conditional effects.^10^ ^–^ ^13^ The result is that conditioning on a covariate that is prognostic of outcome in the analysis model moves the treatment effect estimate and fundamentally changes its interpretation, even without interaction or effect modification.

An estimand defines the exact treatment effect of interest; the statistical method used to estimate the estimand is an estimator, and the numerical value computed by the estimator is an estimate. To date, there has been discussion and disagreement in the literature over whether population-average conditional or marginal estimands are more suitable for population-level decision-making based on effectiveness—including between the authors of this article. Some of the authors have previously argued that targeting the conditional estimand is more desirable, due to increased power to detect treatment effects and differences, resulting in a more distinct ranking of treatments^14^; others have previously argued that the population-average marginal estimand should always be targeted for population-level decision-making, and that the target estimand should be selected based on its relevance to the research question of interest and the decision-making problem.^15^ However, both of these arguments so far have not recognised a fundamental issue: the conditional and marginal estimands correspond to two distinct decision questions that are not aligned when effect modification is present and may give a different ranking of treatments.

In this article, we aim to clarify the different estimands, their interpretation, and implications for decision making on the basis of effectiveness and cost-effectiveness. We focus primarily on the context of population-adjusted indirect comparisons and evidence syntheses, although the arguments apply equally to any context where non-collapsible effect measures are used and effect modification is present, including the analyses of single RCTs and generalising/transporting RCTs to target populations. We begin by setting out terminology and defining conditional and marginal estimands for non-collapsible effect measures with a binary outcome. We describe a range of current population adjustment approaches and the estimands that they target. Using a worked example, we then demonstrate the conflict between population-average conditional and marginal estimands when there is effect modification, which we interpret in the context of decision-making. We then make recommendations for decision-making in the presence of effect modification, before concluding with a discussion.

## 2 Defining conditional and marginal estimands

Consider a binary outcome that occurs with event probability 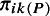 for an individual *i* receiving treatment 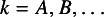 in population *P* with covariates 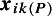 , under the following model: 
(1)

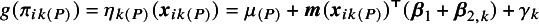

where 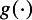 is a link function that transforms probabilities onto the linear predictor 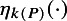 , 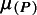 is the intercept (baseline risk), 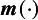 is a function of the covariates, 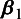 are prognostic effects, 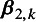 are effect-modifying interactions for treatment *k*, and 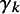 is the individual-level conditional treatment effect for an individual with 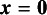 . We set 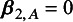 and 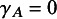 .

Non-collapsibility depends on the choice of link function 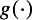 , and in particular on whether the function 
(2)

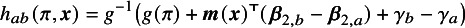

is linear in 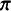 .^12^ ^,^ ^13^ The function 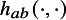 , maps the event probabilities on one treatment *a* to event probabilities on another treatment *b*. Daniel et al.^13^ term 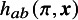 the characteristic collapsibility function (CCF), and consider the case where there is no effect modification ( 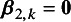 for all *k*) so the CCF no longer depends on the covariates 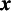 . When the CCF is not linear in 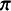 , the corresponding effect measure is not collapsible; this is the case for example when 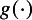 is the logit or probit link function, which correspond to log odds ratios or probit differences. When the CCF is linear in 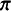 , for example when 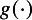 is the identity or log link function, the corresponding effect measure (risk differences or log risk ratios, respectively) will be collapsible. However, non-collapsibility is a necessary consequence of probabilities being bounded between 0 and 1: modelling collapsible effect measures directly can result in predictions outside of this range, and induces purely mathematical treatment-covariate interactions to avoid impossible predictions.

There are several different potential estimands that may be of interest, and these do not typically coincide for non-collapsible effect measures such as log odds ratios, even in the absence of effect modification.

The individual-level conditional treatment effects between each pair of treatments *b* vs. *a* for an individual with covariates 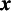 are given by the difference in the linear predictors on each treatment: 
(3)

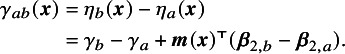

This estimand has an individual-specific interpretation, provided that relevant sources of subject-level heterogeneity are accounted for, and depends on the specific values of any effect modifiers for an individual. However, whilst these individual-level conditional treatment effects may be of interest to individual patients, these are not typically the focus for population-level decision making, which is instead concerned with population-average estimands. A related estimand is the individual-level conditional effect at the mean covariate values, 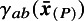 , where 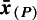 is the mean of 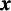 in the population *P*. Whilst estimates of this estimand are sometimes reported, their interpretation is problematic, especially with discrete covariates since it is impossible for an individual to have the “average” value; the individual-level conditional effect at the mean is therefore not useful for decision-making.

Population-average conditional treatment effects between each pair of treatments *a* and *b* in population *P* are obtained by averaging the individual-level treatment effects over the covariate distribution in the population on the linear predictor scale: 
(4)

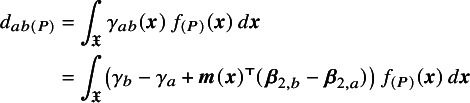

where 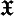 is the support of 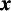 and 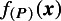 is the joint distribution of 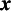 in the population. The population-average conditional treatment effects 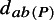 can be interpreted as the average of the individual-level treatment effects in the population. Calculating (4) requires information on the distribution of effect-modifying covariates in the population *P*. In the common special case where 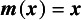 , the covariate means 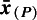 are sufficient to calculate (4) since the linear predictor is linear in the covariates and the integral simplifies to 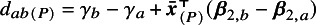 , where 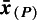 is the mean of 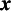 in the population *P*.

The individual-level conditional event probabilities on each treatment, for an individual with covariates 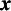 , are given by back-transforming the linear predictor onto the probability scale: 
(5)

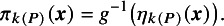

Again, as with the individual-level conditional treatment effects 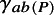 , the individual-level conditional event probabilities are relevant to specific individuals and are not typically the focus for population-level decision making.

Population-average marginal treatment effects are obtained as a summary of the average event probabilities on each treatment: 
(6)

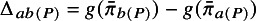

where 
(7)

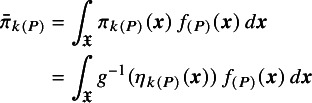

is the average event probability on each treatment. The population-average marginal treatment effects 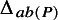 can be interpreted in terms of the effect of treatment on the average event probabilities in the population. Calculating (5), (6), and (7) requires information on the baseline risk 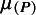 in the population *P* (see Section 7 for practical considerations); (6) and (7) also require information on the joint covariate distribution 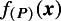 in the population *P*.

Here we have defined the estimands in terms of a generative outcome model. These estimands can also be defined in terms of potential outcomes without reference to any model; we give definitions in the potential outcomes framework in Appendix A. We note that, although the estimands can be defined in a “model-free” manner, all current population adjustment methods with limited IPD will impose an assumed outcome model of the form (1) in order to form an indirect comparison, either explicitly (e.g., STC, ML-NMR) or implicitly (e.g., MAIC).^16^

The terms “population-average” and “marginal” are often used interchangeably, but here we make a conceptual distinction. Population-average refers to a quantity that has been averaged over the population, which may be conditional (like 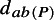 ) or marginal (like 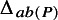 ), whereas marginal refers to the scale on which this averaging has taken place (i.e., the probability scale, rather than the linear predictor scale for conditional quantities). Similarly, it is sometimes said that the population-average marginal effect is “the effect” of moving a population from one treatment to another. However, we clearly see that this intervention effect can be defined in multiple ways, depending on the scale on which the average is taken. The population-average marginal estimand 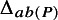 averages the counterfactual event probabilities on each treatment and compares them, whereas the population-average conditional estimand 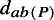 averages the individual counterfactual treatment effects.

For population-level decision-making, often the decision is made based on cost-effectiveness rather than purely effectiveness, such as in health technology assessment. In this case, the estimands above are not of direct interest, but are instead considered inputs to a cost-effectiveness model that evaluates the expected net benefit on each treatment. For now, we consider only effectiveness decisions based on 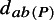 or 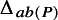 , and we revisit cost-effectiveness decisions in Section 5.

## 3 Population adjustment methods

In an ideal scenario, IPD would be available from every study, in which case the “gold standard” approach is an IPD network meta-regression which accounts for differences in effect modifiers between studies and can produce population-average conditional or marginal treatment effect estimates in any population of interest (including external target populations) via equations (4) and (6).^17^ ^–^ ^20^ However, this scenario is uncommon in many practical applications, for example in health technology assessment where a company making a submission to an agency such as the National Institute for Health and Care Excellence in England has IPD from their own study or studies, but only published aggregate data from their competitors’. Population adjustment methods are designed with this limited-IPD scenario in mind, and aim to use IPD available from a subset of studies to account for differences in the distribution of effect modifiers between studies.^16^ ^,^ ^21^ Different population adjustment methods target different estimands, and produce estimates that are relevant to different target populations; these are summarised in Table 1.

**Table 1:** Evidence synthesis and population adjustment methods, and the estimands and target populations targeted by each

Method	Estimand	Target population
(Network) meta-analysis or indirect comparison	Marginal	Average/common population of all included studies
IPD network meta-regression	Conditional or marginal	Any
ML-NMR	Conditional or marginal	Any
MAIC	Marginal	Single aggregate study
STC (plug-in means)	Neither, typically combines incompatible conditional and marginal estimates	Single aggregate study
STC (simulation)	Marginal	Single aggregate study
STC (G-computation)	Marginal	Single aggregate study
NMI	Neither, typically combines incompatible conditional and marginal estimates	Any

*Note:* Abbreviations: IPD, individual patient data; ML-NMR, multilevel network meta-regression; MAIC, matching-adjusted indirect comparison; STC, simulated treatment comparision; NMI, network meta-interpolation.

ML-NMR is a generalisation of the IPD network meta-regression framework to incorporate aggregate data, by integrating the individual-level model over each aggregate study population (as in equation (7)).^5^ This approach avoids aggregation bias, unlike approaches to combining IPD and aggregate data in network meta-regression that simply “plug in” mean covariate values for the aggregate studies into the individual-level model.^22^ ^–^ ^24^ ML-NMR combines evidence at the level of the individual conditional treatment effects, and can be used to produce estimates of both conditional and marginal estimands following equations (4) and (6), in any target population of interest.^5^ ^,^ ^14^ The marginalisation integrals (7) for each aggregate study are typically calculated using efficient quasi-Monte Carlo numerical integration, which can also be used to produce estimates for external target populations with a given covariate distribution.^5^ The multinma R package implements ML-NMR models for a range of outcome types, as well as full-IPD and aggregate data only network meta-analysis as special cases, and provides functionality to produce estimates of all of the different estimands defined in Section 2.^25^

MAIC is a weighting approach, where the method of moments is used to estimate weights that match covariate means or higher order moments in an IPD study to those reported in an aggregate study.^6^ MAIC targets the population-average marginal estimand (6), but since no conditional regression model is fitted this bypasses the need to evaluate any marginalisation integral (equation (7)) entirely. MAIC can only produce estimates relevant to the aggregate study population in a two-study indirect comparison.^16^

STC is a regression adjustment approach that fits a regression model in an IPD study and uses this to predict outcomes in an aggregate study population.^7^ The most common form of STC typically combines a conditional estimate from the IPD study, obtained by plugging-in mean covariate values to equation (3), with a marginal estimate as reported by the aggregate study, which are incompatible and is thus biased against both the population-average conditional (4) and marginal (6) estimands.^14^ ^,^ ^15^ Plug-in means STC should therefore be avoided. Other forms of STC are available that avoid this problem and target the population-average marginal estimand via simulation (to evaluate equations (6) and (7)), however these incur additional sampling variation by trying to simulate a limited number of participants in the aggregate trial.^7^ All forms of STC can only produce estimates relevant to the aggregate study population in a two-study indirect comparison.^16^

A more sophisticated form of STC based on G-computation addresses several of the issues with other forms of STC.^9^ G-computation STC targets the population-average marginal estimand (6), fitting a regression model in an IPD study, and evaluating the marginalisation integral (7) over an aggregate study population using simulation (parametric G-computation). Uncertainty is fully quantified by implementing the approach in a Bayesian framework.^9^ However, like other forms of STC, this approach can only produce estimates relevant to the aggregate study population in a two-study indirect comparison.^9^ Similar simulation-based STC approaches have recently been published.^26^ ^,^ ^27^

The key assumption made by all population adjustment methods in a connected network of comparisons (so-called *anchored* population-adjusted indirect comparisons) is *conditional constancy of relative effects*.^16^ This requires all effect modifiers to be known and appropriately adjusted for, such that 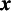 includes all effect modifiers and their functional form 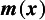 in the outcome model (1) is correctly specified. For regression-based approaches like ML-NMR and STC, this model specification is explicit. For MAIC, the choice of covariate moments to use for matching implies the form of the underlying outcome model (1); for example, matching only on covariate means leads to 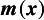 being linear in 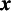 . In a disconnected network or with single-arm studies, *unanchored* population-adjusted indirect comparisons may be created, relying on the assumption of *conditional constancy of absolute effects*. This is a much stronger assumption, which requires producing accurate predictions of absolute outcomes accross populations, and is widely considered very difficult to meet.^16^

Population adjustment analyses are often required to make simplifying assumptions for identifiability, most commonly invoking the *shared effect modifier assumption*, which states that effect modifier interactions are equal for a set of treatments; that is 
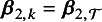
 for all treatments *k* in the set 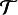 . This assumption may be reasonable if treatments in 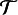 are from the same class and share a mode of action, otherwise this assumption is unlikely to hold; this needs to be considered on a case-by-case basis.^16^ For MAIC and STC, with a continuous outcome and a linear outcome model, making the shared effect modifier assumption for treatments *B* and *C* means that the estimated relative treatment effect 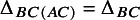 is constant across populations, and can be applied in any target population and not just the 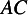 trial population. For other outcomes and outcome models, however, this assumption is not sufficient to make the marginal effect 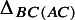 transportable, as this marginal effect is specific to the distribution of covariates and baseline risk in the 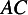 population. For ML-NMR, the shared effect modifier assumption may be used in smaller networks to identify the model in the absence of sufficient data, for example in a two-study indirect comparison.^5^ ML-NMR can use this assumption regardless of the outcome type or outcome model, since it is applied to the individual-level conditional outcome model. If this assumption does not hold and the model cannot be identified via other means (e.g., external information to inform prior distributions for the interactions or other structural assumptions about interactions), then ML-NMR is limited to producing estimates in the aggregate study population, like MAIC and STC. In even moderately-sized networks, ML-NMR may allow the shared effect modifier assumption to be assessed or removed entirely.^28^ We examine the shared effect modifier assumption further in Section 4.3.

Network meta-interpolation (NMI) is different to other regression-based approaches; whilst an outcome regression model is defined, this model is not estimated directly. Instead, published subgroup analyses from each trial are “interpolated” to a specific target population by solving equations based on the best linear unbiased predictor, and then these adjusted estimates are combined in a standard NMA.^29^ The motivation of NMI is to produce population-adjusted estimates without making the shared effect modifier assumption, by using additional information in the form of subgroup analyses from all studies.^5^ ^,^ ^16^ However, NMI incurs similar biases to plug-in means STC, as the reported study-level treatment effect estimates in trial publications are unlikely to be compatible with the conditional treatment effect estimates required by NMI to perform interpolation. NMI therefore typically mixes incompatible estimates within the model (within studies, as opposed to across studies for plug-in means STC), and is thus biased against both the conditional and marginal estimands. Furthermore, since the outcome regression model is not fully estimated, it is not possible for NMI to produce population-average marginal treatment effects or absolute predictions (e.g., average event probabilities). In certain specific scenarios (i.e., binary covariates and linear outcome models) NMI does appear promising; however, the properties and performance of NMI are not yet fully understood, and simulation studies have not directly investigated bias or adequacy of variance estimation.

Standard network meta-analysis, pairwise meta-analysis, and indirect comparison methods combine aggregate data from each study without adjusting for covariates, targeting a population-average marginal estimand. The constancy of relative effects assumption is required, meaning that there is (on average) no imbalance in effect modifiers between populations. Balanace in baseline risk and prognostic factors is also required unless the outcome is continuous and the outcome generating model is linear in the covariates, as these also modify the marginal effect estimates. The target population is an average of the included study populations, which are typically assumed to all be representative of a single common population.

## 4 Example

To illustrate the issues that arise with non-collapsible effect measures when there is effect modification, we consider a simple example with a single covariate *x* that is both prognostic of outcome and effect modifying, uniformly distributed in the population between 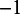 and 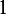 , and binary outcomes on three treatments 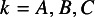 that are modelled on the log odds ratio scale using Equation (1) with the logit link function 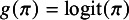 and 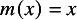 .

### 4.1 Effect modification can result in conflicting rankings

It is well-understood that the magnitudes of population-average conditional and marginal effects 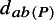 and 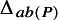 will typically differ, even in the absence of effect modification.^10^ ^–^ ^13^ However, when there is effect modification the direction of effect can also differ between 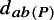 and 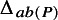 , resulting in conflicting treatment rankings. It is straightforward to find values of the coefficients in Equation (1) where this occurs, for example 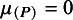 , 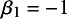 , 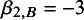 , 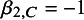 , 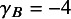 , 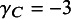 . This results in population-average conditional treatment effects 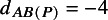 and 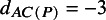 (i.e., *B* better than *C* for reducing a harmful outcome), but population-average marginal treatment effects 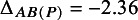 and 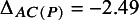 (i.e., *C* better than *B*). This is because treatment *C* results in a lower average event probability overall: 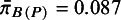 , 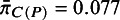 (and 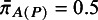 ).

**Figure 1:**
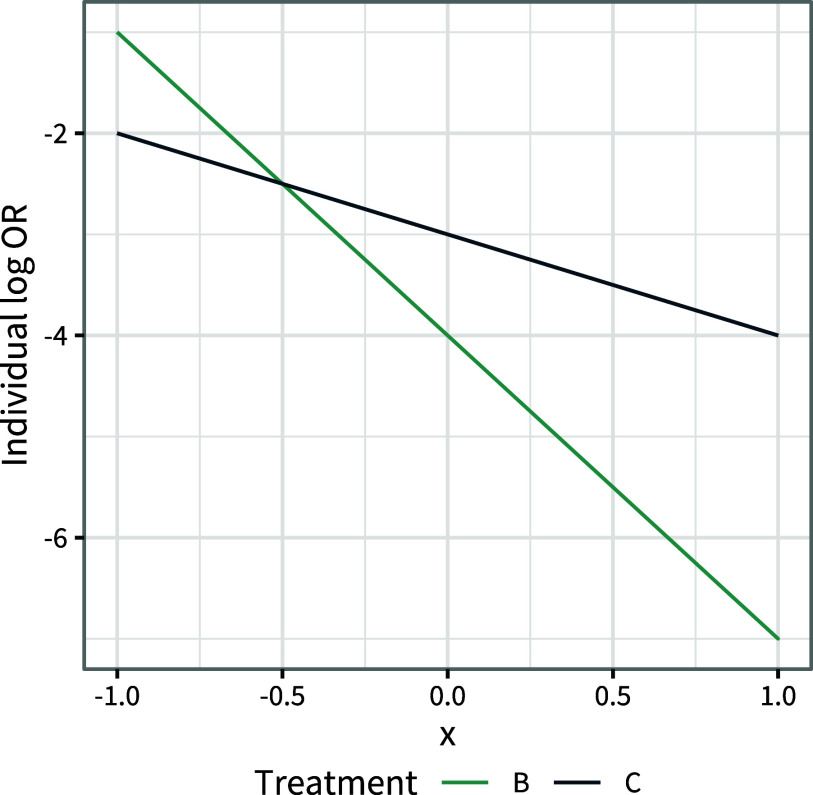
Individual-level log odds ratios 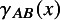 and 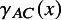 for treatments *B* and *C* compared to *A*, over the range of the covariate *x* in the population.

Basing a decision on these population-average marginal treatment effects would result in choosing treatment *C*, but 75% of the population are given an inferior treatment and are expected to do better on treatment *B* (Figure 1). On the other hand, basing a decision on these population-average conditional treatment effects would result in choosing treatment *B*, but a lower expected number of events 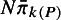 (in a population of size *N*) would be achieved by treatment *C*.

### 4.2 Marginal ranks depend on baseline risk and prognostic factors

**Figure 2:**
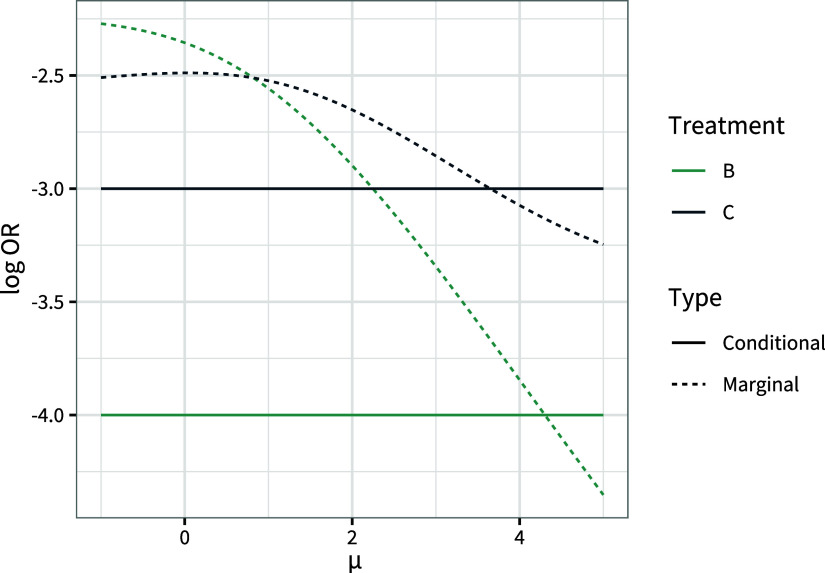
Population-average marginal ( 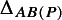 and 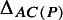 ) and conditional ( 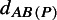 and 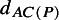 ) log odds ratios for treatments *B* and *C* compared to *A*, for a range of values of the baseline risk 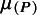 .

Furthermore, the population-average marginal treatment effects and rankings are dependent on the baseline risk 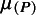 , as well as the distribution of the covariate *x* (even if it is only prognostic), but the population-average conditional treatment effects and rankings only change when the distribution of effect-modifying covariates changes. Figure 2 shows the population-average conditional and marginal treatment effects for a range of values of the baseline risk 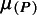 . The population-average conditional treatment effects 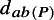 are constant over all values of the baseline risk, but the population-average marginal treatment effects 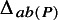 change depending on the baseline risk and switch ranks. Also note that the widely-known result that conditional effects are always further from the null than marginal effects^12^ ^,^ ^13^ does *not* hold when there is effect modification; here the marginal log odds ratio for treatment *C* is further from the null for 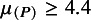 . Consequently, the corollary result that power is always greater for conditional effects also does not hold when there is effect modification.

**Figure 3:**
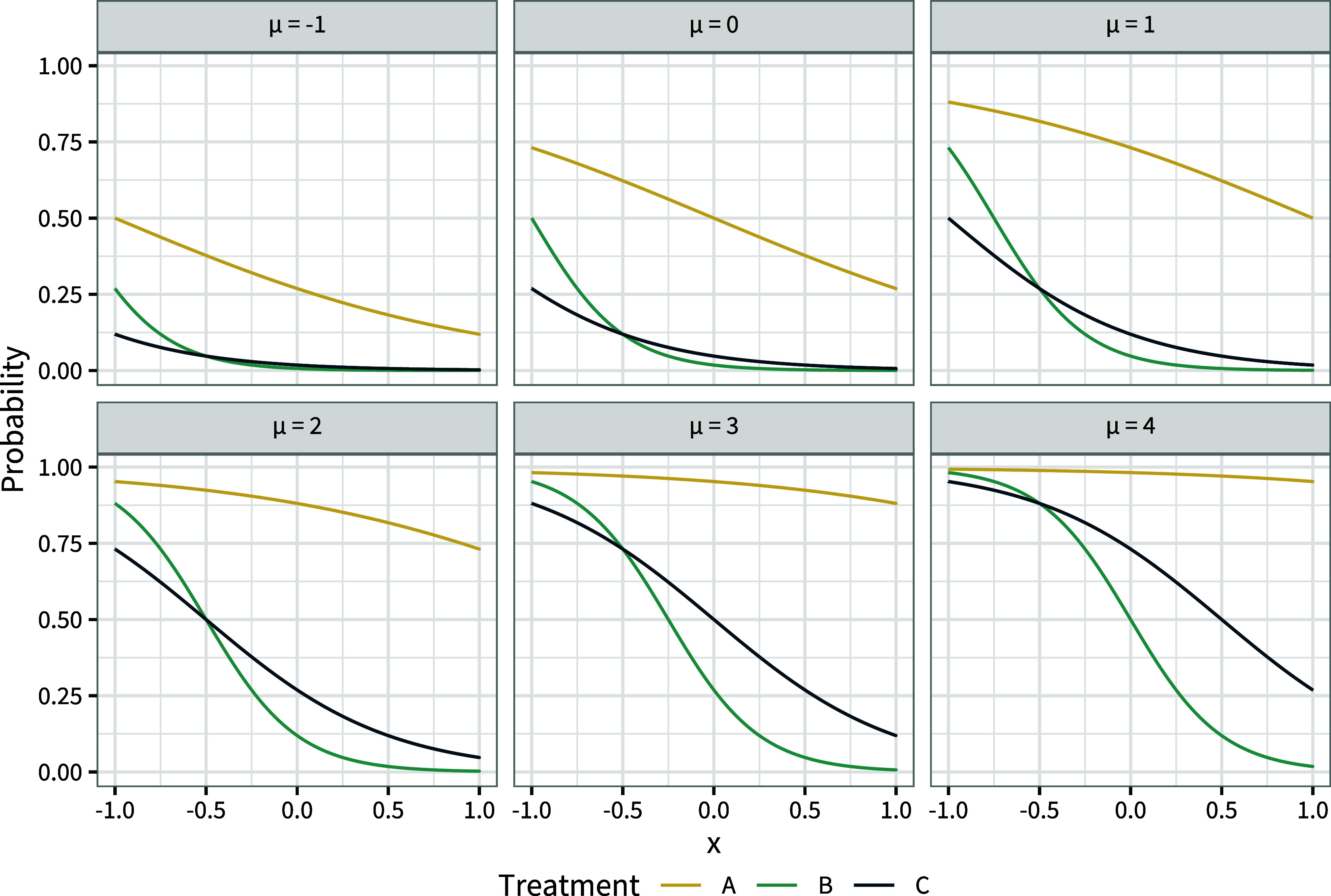
Individual event probabilities 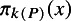 on each treatment over the range of covariate values *x* in the population, for a range of values of the baseline risk 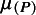 .

The population-average marginal effects change because changing the baseline risk changes the individual event probabilities (Figure 3), which are averaged over the population to obtain 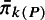 and the marginal effects.

### 4.3 The shared effect modifier assumption

**Figure 4:**
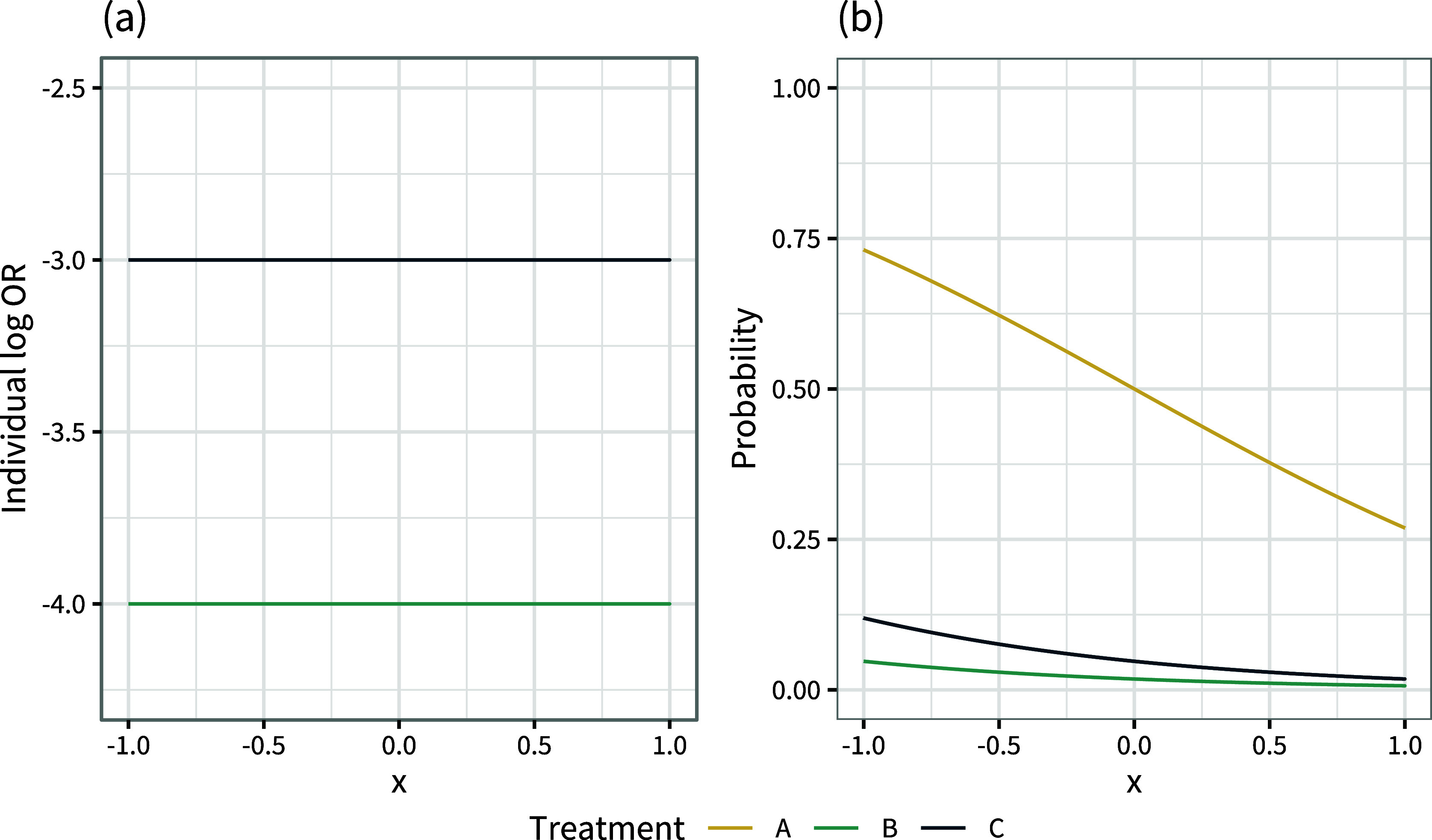
Individual-level log odds ratios 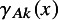 (a) and event probabilities 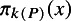 (b) when there is no effect modification ( 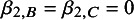 ), over the range of the covariate *x* in the population.

We see in Figure 3 that the curves of individual-level event probabilities 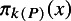 by covariate *x* on each treatment intersect. This crossing of event probability curves due to effect modification is the reason that the conditional and marginal treatment effects can give different rankings. If there is no effect modification then the individual-level log odds ratios between all treatments are constant (Figure 4a), the event probability curves cannot cross (Figure 4b), and the conditional and marginal rankings and decision questions are aligned.

**Figure 5:**
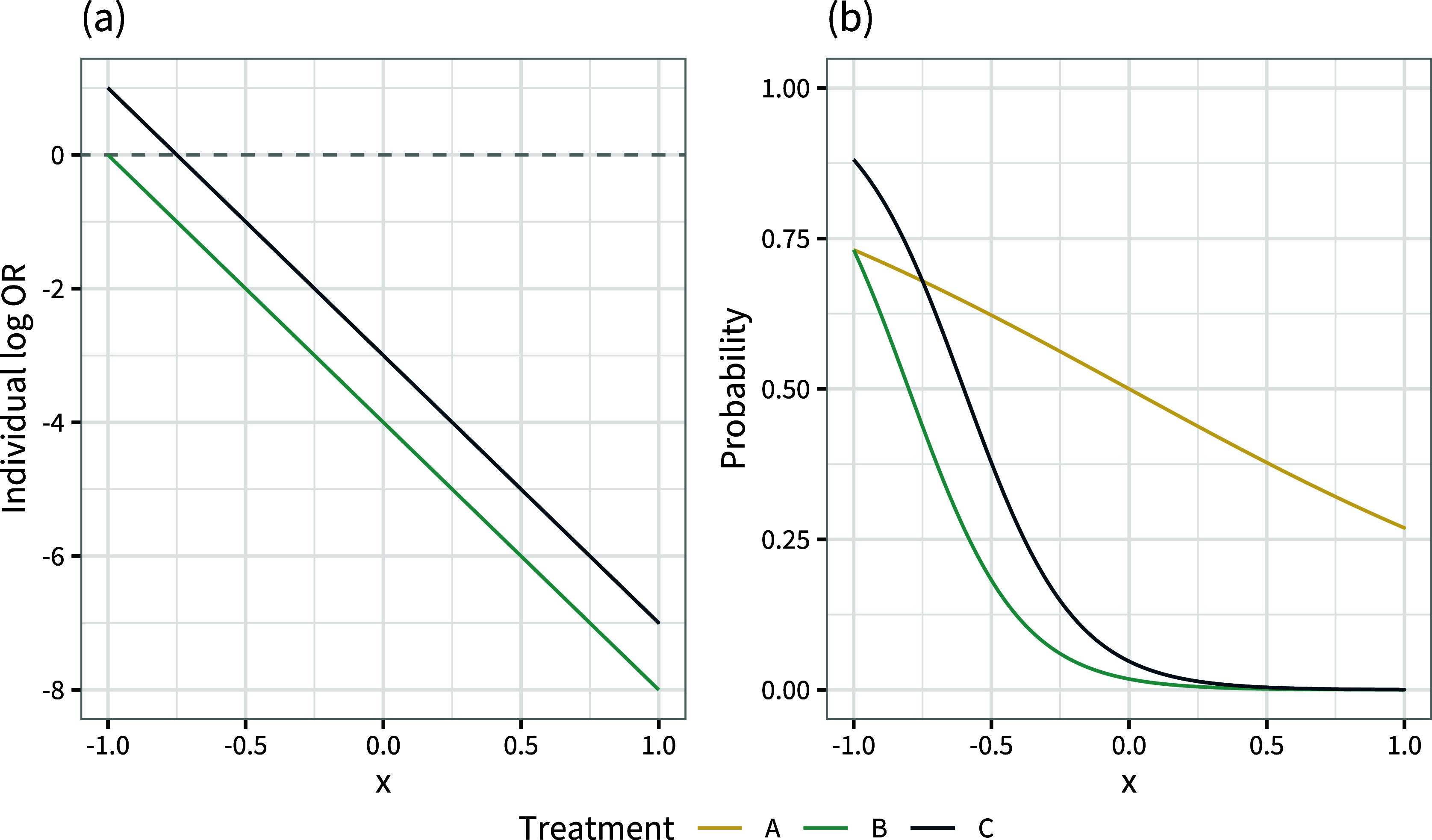
Individual-level log odds ratios 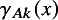 (a) and event probabilities 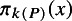 (b) when the shared effect modifier assumption is made for treatments *B* and *C* ( 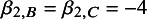 ), over the range of the covariate *x* in the population.

Moreover, the individual event probability curves of two treatments cannot cross if the effect modifier interaction coefficients are the same for these two treatments; for example, if 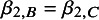 (Figure 5b). In this situation the individual-level odds ratio between these two treatments is again constant (the curves in Figure 5a are parallel). The assumption that effect modifier interaction coefficients are the same for a set of treatments is called the shared effect modifier assumption (see Section 3).^16^ ^,^ ^21^ The shared effect modifier assumption may sometimes be used for ML-NMR when there are insufficient data to estimate separate interaction terms for each treatment, for example in a two-study indirect comparison, in order to produce estimates for populations other than the aggregate study population.^5^ Pairs of treatments between which the shared effect modifier assumption is not made can have individual odds ratios and event probability curves that cross; in Figure 5a the individual odds ratio for *C* (compared to *A*) intersects the line of no effect, and as a result the event probability curves for *A* and *C* cross. This means that, even if the shared effect modifier assumption is made for treatments *B* and *C*, the marginal ranking of treatment *A* can still change. With a continuous outcome and linear outcome model, the shared effect modifier assumption can also be used by MAIC and STC in order to produce relative effect estimates that are relevant to a target population other than that of the aggregate study in the indirect comparison, since then 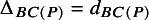 is constant across all populations.^16^ In all other cases, the population-average marginal treatment effects 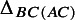 produced by MAIC and STC (with simulation) are specific to the distributions of covariates and baseline risk in the aggregate 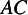 study population, and cannot be transported to another population with different distributions of covariates or baseline risk.

### 4.4 An example with a binary covariate in a contingency table

To help further solidify these ideas, we now consider an example with a binary outcome and a single binary covariate through a contingency table. Consider a trial of four treatments *A*, *B*, *C*, and *D*, randomised equally, in a population where the prevalence of a biomarker *x* is 25%. This biomarker is prognostic and effect-modifying, and we are interested in reducing occurrence of some harmful event. Table 2 shows the numbers of individuals who did and did not experience the event, within subgroups defined by the biomarker *x* and over the whole population.

**Table 2:** Contingency table for an illustrative example of four treatments, stratified by a biomarker x

	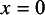		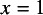		Overall population	
Treatment	Events	Non-events	Events	Non-events	Events	Non-events
*A*	202	548	156	94	358	642
*B*	89	661	42	208	131	869
Marginal OR					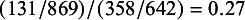	
Conditional OR	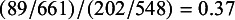		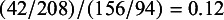		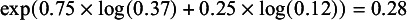	
*C*	13	737	144	106	157	843
Marginal OR					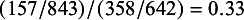	
Conditional OR	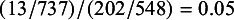		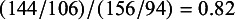		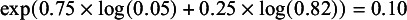	
*D*	137	613	6	244	143	857
Marginal OR					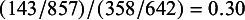	
Conditional OR	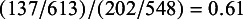		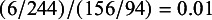		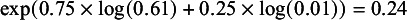	

*Note:* Population-average marginal odds ratios, subgroup-specific conditional odds ratios, and population-average conditional odds ratios are calculated vs. treatment *A*.

In Table 2, we then calculate the population-average marginal odds ratios, subgroup-specific conditional odds ratios, and population-average conditional odds ratios for each treatment compared to *A*. Here, since we have a binary covariate, calculating the population-average conditional odds ratios via the integral in equation (4) simplifies to taking the weighted average (on the log odds ratio scale) of the subgroup-specific conditional odds ratios according to the prevalence of the biomarker in the population. Based on the population-average marginal odds ratios, treatment *B* is the best, as it results in the lowest number of events overall. However, the population-average conditional odds ratios give a different ranking: treatments *C* and *D* are both ranked better than *B*, with treatment *C* being the best.

Treatment *C* is the most effective treatment for most individuals in this population, i.e., in the biomarker-negative subgroup ( 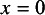 ) which makes up 75% of the population. This leads to *C* having the best population-average conditional odds ratio. However, *C* is less effective than *B* for the smaller biomarker-positive subgroup ( 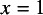 ); the increased number of events in this subgroup result in a higher number of events overall on treatment *C* than *B*, and hence a worse population-average marginal odds ratio.

Treatment *D* is less effective than *B* for most of this population—the biomarker-negative subgroup ( 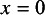 )—and has a higher event rate overall. The population-average marginal odds ratio for treatment *D* is therefore worse than for treatment *B*. However, *D* is highly effective for the biomarker-positive subgroup ( 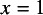 ), to an extent that is sufficient to give a better population-average conditional odds ratio than treatment *B*.

We see here how the population-average conditional and marginal effects weigh up effectiveness over the population differently. The population-average marginal effects weigh up the expected number of events overall, i.e., the average is taken on the probability scale. The population-average conditional effects weigh up the expected individual or subgroup effectiveness over the population, i.e., the average is taken on the additive linear predictor scale.

As shown earlier in Section 4.1, we again see here that the treatment with the best population-average marginal effect (*B*) is not always the best treatment for the majority of individuals when effect modification is present. Furthermore, when covariates are discrete or non-symmetrically distributed, or treatment or covariate effects are non-linear, the treatment with the best population-average conditional effect is not always the best treatment for the majority of individuals. In this example with a binary covariate, treatment *D* has a better population-average conditional effect than treatment *B*, but *D* is less effective than *B* for most individuals.

Selecting the single treatment *B* with the best population-average marginal effect results in a decision that minimises the number of events overall. However, the rank conflict with the population-average conditional effects indicates that there is substantive differential effectiveness within the population, and a decision stratified by subgroup may be closer to optimal. In this case, treatment *B* is inferior for every individual in the population: treating biomarker-negative individuals with *C* and biomarker-positive individuals with *D* is the optimal decision. This stratified treatment decision would result in the least number of events overall, a population-average marginal odds ratio of 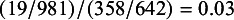 , and a population-average conditional odds ratio of 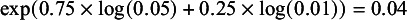 .

## 5 Interpretation

Rank-switching between the population-average conditional and marginal effects can only occur in the presence of effect modification, between treatments that have different interaction terms (i.e., no shared effect modifier assumption), and when this causes treatment ranks to change across individuals/subgroups in the population. We give a formal proof of this statement in Appendix B. However to see this intuitively, consider that rankings based on the population-average marginal treatment effects 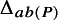 can only change compared to the population-average conditional treatment effects 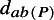 if the individual event probabilities on each treatment 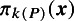 change ranks within the population (i.e., if the event probability curves cross as in Figure 3). This can happen if and only if the individual-level treatment effects 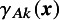 change ranks within the population (i.e., if the individual treatment effect curves cross as in Figure 1), which can happen if and only if there is effect modification between the two treatments.

It is well-understood that population-average marginal treatment effects are population-specific, and depend on the distributions of baseline risk and prognostic factors, as well as any effect modifiers. However, when there is effect modification we have seen that the marginal treatment rankings can also change—even if the only factors that change are those that do not affect individual treatment effects (baseline risk, prognostic factors). Conditional population-average treatment effects and rankings will only change depending on the distribution of effect modifiers, and do not depend on baseline risk or prognostic factors.

The population-average conditional and marginal effects have different interpretations, and correspond to different decision questions regarding effectiveness. The population-average conditional treatment effects represent the average of the individual treatment effects experienced in the population. They answer the question “Which treatment has the greatest effect for individuals, on average, in this population?” The population-average marginal treatment effects (whether odds ratios, risk ratios, or risk differences) quantify effectiveness in terms of the average event probabilities on each treatment, and for non-collapsible effect measures, by definition, do not represent any average of the individual or subgroup treatment effects experienced in the population, even without effect modification. They answer the question “Which treatment minimises (or maximises) the marginal average event probability in this population?”

These two decision questions are equivalent *except* when there is effect modification. When there is effect modification and the individual treatment effects cross within the population, the two can give different rankings. As a result, a decision based on population-average marginal treatment effects to obtain the minimum (or maximum) average event probability can result in choosing a treatment that is inferior for the majority of individuals in the population. Conversely, a decision based on population-average conditional treatment effects to obtain the most effective treatment on average for each individual in the population can result in choosing a treatment that gives a higher (or lower) average event probability than another treatment option.

Either of these decision questions and estimands might be justified. Basing a decision on the population-average conditional effects means that decision-makers want to maximise the benefit, on average, for each individual in the population. Basing a decision on the population-average marginal effects means that decision-makers want to minimise (or maximise) the expected number of events over the population, for example if (non-)events have a high associated cost or disutility. Indeed, in many cases decision-makers may wish to satisfy both decision questions; however, when there is effect modification there may not be a single treatment that achieves this over the entire population.

For cost-effectiveness decisions, neither of the above decision questions or estimands are of direct interest. Instead, the decision question is “Which treatment maximises the expected net benefit in this population?”, and the relevant quantity is the expected net benefit (NB) on each treatment 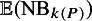 . The net benefit is typically a function 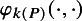 of the average event probabilities 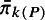 , or the individual event probabilities 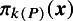 for the distribution of the covariates in the population, as well as other parameters 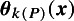 such as resource use costs and adverse events which may also vary over the population. When there is patient heterogeneity, such as that caused by effect modification, a cost-effectiveness analysis should handle this appropriately by averaging (integrating) net benefit over the population, which necessitates constructing the net benefit as a function of the individual event probabilities 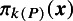 ^30^: 
(8)

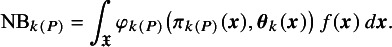

In simple cases this integral may be evaluated directly, however discrete event simulation is often used instead to construct and evaluate such cost-effectiveness models.^31^ ^,^ ^32^ Comparing equation (8) with equations (4) and (7), we see that the population-average conditional estimand corresponds to a net benefit function that is linear in individual treatment effects 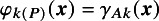 and the population-average marginal estimand corresponds to a net benefit function that is linear in individual event probabilities 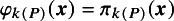 , when effectiveness is the only consideration.

## 6 Considerations for survival outcomes

Similar arguments can be applied to survival or time-to-event outcomes analysed using proportional hazards models, since hazard ratios are also non-collapsible. Consider a general proportional hazards model where the hazard function for an individual *i* receiving treatment *k* in population *P* with covariates 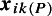 at time *t* is
(9a)

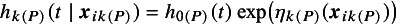



(9b)

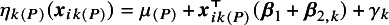



for some baseline hazard function 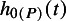 , with corresponding survival function 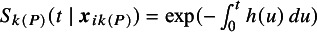 .

The population-average marginal hazard ratio 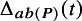 is 
(10)

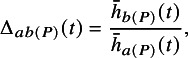

the ratio of the marginal hazard functions 
(11)

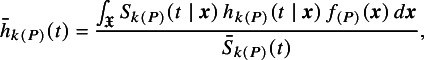

where 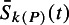 is the population-average marginal survival function (or standardised survival function) 
(12)

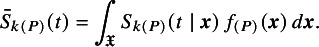

The marginal hazard function 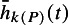 can be considered a weighted average of the individual-level hazard functions, weighted by the probability of surviving to time *t*. Notably, the population-average marginal hazard ratio 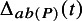 is *time-varying*, as well as depending on the shape of the baseline hazard function 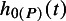 , and the distributions of baseline hazard and all prognostic and effect modifying covariates. This means that, if covariates are present, proportional hazards mathematically cannot hold at the marginal level. This is the case whether the covariates are prognostic or effect modifying; however, an argument analogous to that in Appendix B shows that effect modification is necessary for the marginal hazard functions to cross (assuming that covariates are balanced between arms at baseline, and that the same baseline hazard function 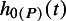 applies in both arms).

The population-average conditional log hazard ratio 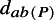 under this model is again given by equation (4)—the average treatment effect over the population, taken on the log hazard scale. Again, 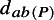 depends only on the distribution of effect modifiers, not on the shape of the baseline hazard function, or the distributions of baseline hazard or prognostic factors, and is not time-varying.

As an example, consider a Weibull proportional hazards model for three treatments, *A*, *B*, and *C*, with a single covariate *x* that is uniformly distributed in the population between 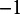 and 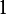 , with survival and hazard functions 
(13a)

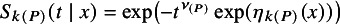



(13b)

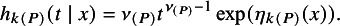



**Figure 6:**
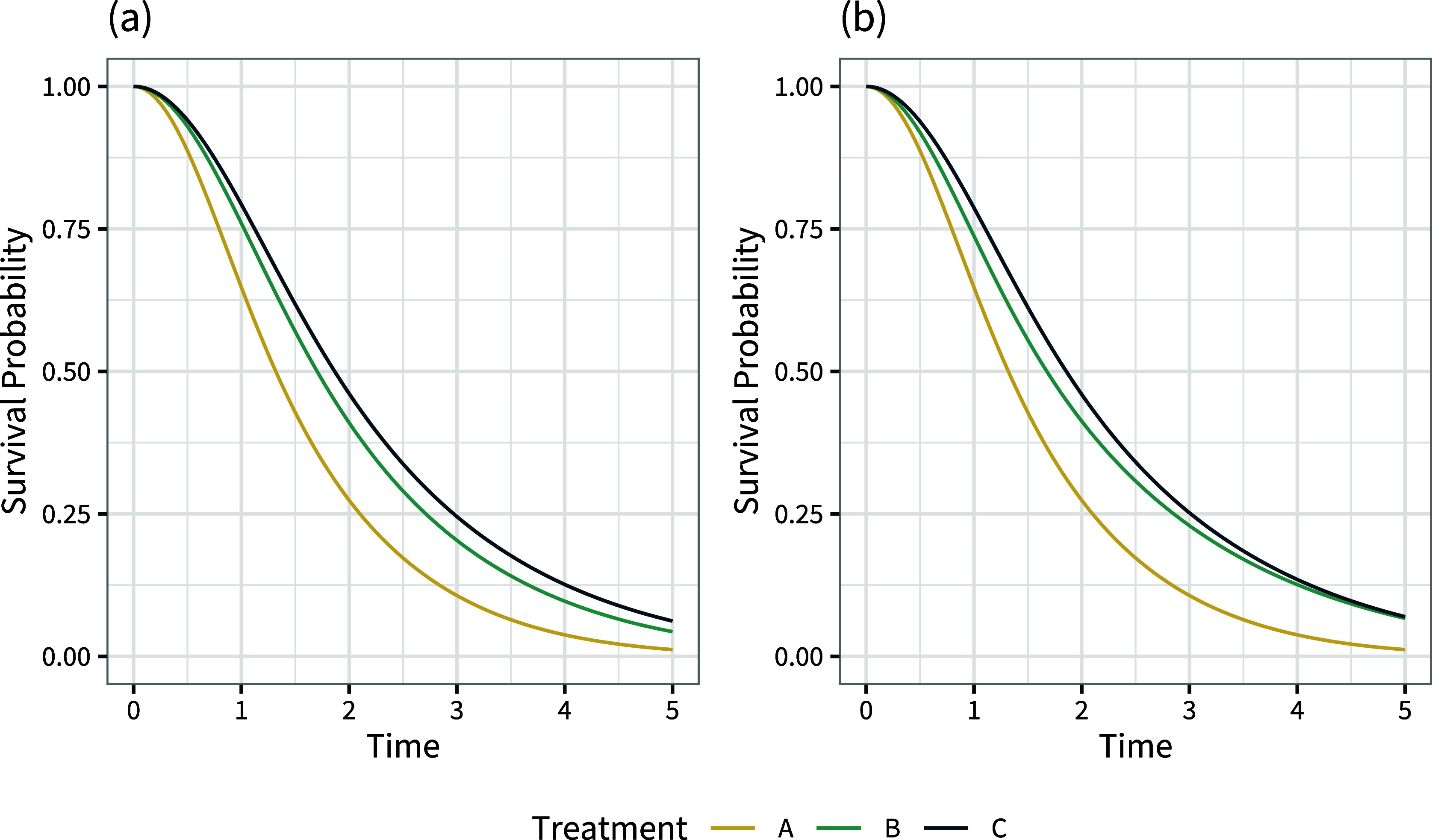
Population-average marginal survival curves with a single uniformly-distributed covariate that is (a) prognostic only, (b) prognostic and effect modifying.

For simplicity, we use a common shape parameter 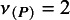 for all three treatments, and set 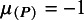 , 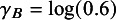 , and 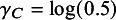 . The covariate *x* we set to be prognostic of survival with 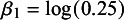 . We then consider two scenarios, one where *x* is only prognostic so 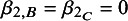 , and the other where *x* is moderately effect modifying, 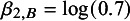 and 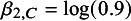 . The population-average marginal survival curves under this set-up are shown in Figure 6.

**Figure 7:**
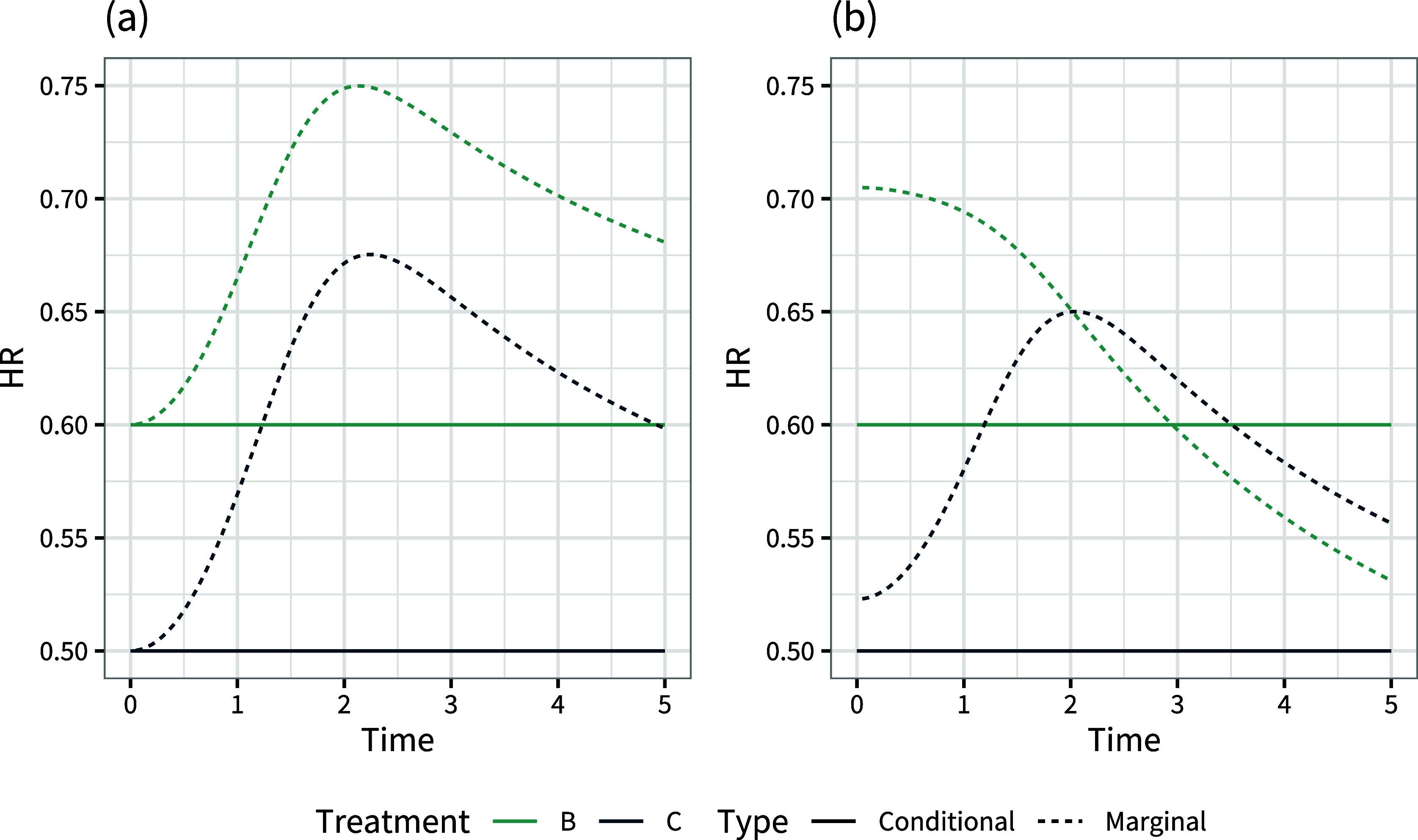
Population-average conditional and marginal hazard ratios vs. treatment *A* over time with a single uniformly-distributed covariate that is (a) prognostic only, (b) prognostic and effect modifying.

**Figure 8:**
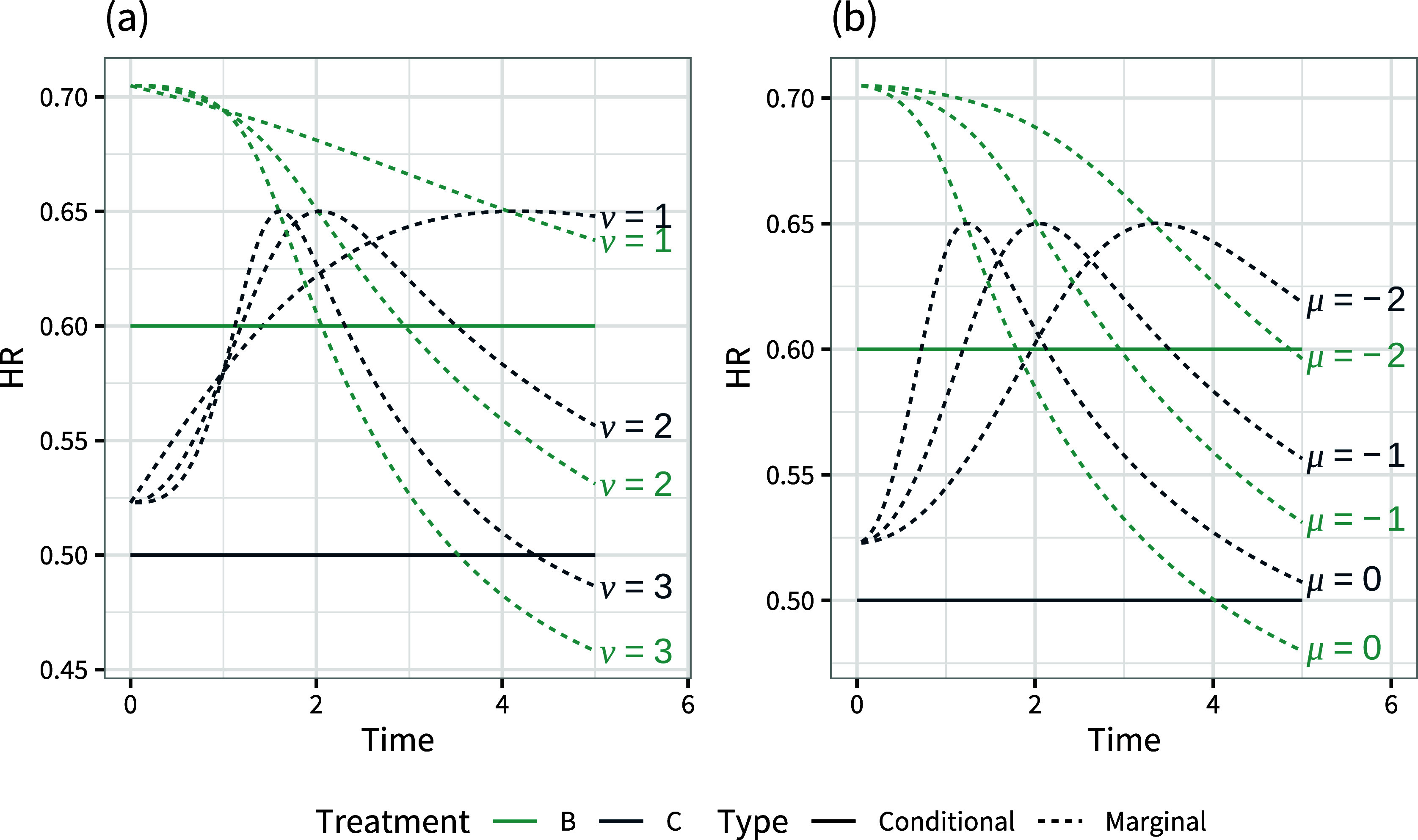
Population-average conditional and marginal hazard ratios vs. treatment *A* over time, varying (a) the shape of the baseline hazard function 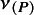 , and (b) the distribution of baseline log hazard 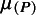 .

The corresponding population-average conditional and marginal hazard ratios are shown in Figure 7. The presence of a prognostic covariate means that the population-average marginal hazard ratios are no longer constant over time; proportional hazards does not hold at the marginal level. When this covariate is also effect modifying, the population-average marginal hazard ratios can also change ranks over time. Figure 8 demonstrates that the population-average marginal hazard ratios depend on the shape of the baseline hazard function and the distribution of baseline hazard, whereas the population-average conditional hazard ratios do not.

With survival outcomes, the key quantities for decision-making are typically the population-average marginal survival functions 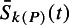 on each treatment and summaries thereof, such as survival probabilities at clinically relevant time points, median survival times, or (restricted) mean survival times. 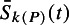 is also the typically the primary input to an economic model for decisions based on cost-effectiveness.

## 7 Recommendations for decision-making in the presence of effect modification

Decision-makers should specify *a priori* the target population and decision question that are of interest, and analysts should ensure that the corresponding estimand is appropriately targeted—be that population-average conditional or marginal estimates for effectiveness decisions, or the necessary inputs to an economic model for cost-effectiveness decisions. In a health technology assessment context, guidance for submissions to the National Institute of Health and Care Excellence (NICE) in England states that the choice of effect modifiers must be pre-specified prior to analysis, clinically plausible, and justified through empirical evidence, expert opinion, or systematic review.^16^ ^,^ ^33^ Similarly, section 4.9 of the NICE health technology evaluation manual expresses a preference for pre-specified identification of subgroups with biological plausibility, and warns against post-hoc “dredging” for subgroup effects.^33^ This applies to subgroups based both on effectiveness (i.e., effect modifiers), and on other factors such as costs, baseline risk, or adverse events. In line with methodological guidance,^16^ it is advisable that: analyses with and without adjustment for effect modifiers be presented, for example standard network meta-analysis alongside a suitable population adjustment method; any modelling assumptions such as choice of covariates to include be explored in sensitivity analyses; and the extent of extrapolation should be considered and estimates treated with caution where extrapolation is required beyond the range of the data.

### 7.1 Decisions based on effectiveness only

For population decision-making based purely on effectiveness, the relevant estimands are population-average conditional or marginal treatment effects in the decision target population, for example after population adjustment. ML-NMR can produce relevant estimates for any decision target population^5^; other approaches like MAIC and STC are limited to producing estimates in the population of the aggregate study in an indirect comparison, which may not represent the decision target population.^16^ In small networks like a two-study indirect comparison, ML-NMR may make use of an identifying assumption such as the shared effect modifier assumption to produce estimates for populations other than the aggregate study population (Section 3). MAIC and STC cannot make use of the shared effect modifier assumption except for continuous outcomes with a linear outcome model, and are restricted to producing estimates for the aggregate study population. Only ML-NMR at present can produce estimates of both conditional and marginal estimands. The population-average conditional and marginal estimands can result in conflicting rankings when there is effect modification, and in some cases a decision-maker may be forced to choose between a treatment that is inferior for the majority of the population or one that results in a worse expected number of events overall. If this is a concern, then the only way to resolve this conflict and realign the two effectiveness decision questions and estimands is to allow different decisions within subgroups based on covariate values.

With just one effect modifier, it is straightforward to visualise the impact on decisions by plotting the individual treatment effects against the covariate (as in Figure 1), but with multiple effect modifiers this quickly becomes infeasible. It is possible to mathematically determine the boundaries between different optimal treatment choices in the *L*-dimensional covariate space, where *L* is the number of effect-modifying covariates. However, this is likely to result in complex decisions that may be difficult to implement and hard to justify.

We propose a more pragmatic approach, where decision-makers consider both the population-average conditional and marginal treatment effects. If the respective rankings agree, then there is no substantial conflict to resolve and a single decision might be justified for the entire population. This does not rule out the possibility that smaller subgroups might obtain greater treatment benefit or lower (or higher) average event probabilities from a different decision, but it does mean that the overall decision is both the most effective on average for each individual in the population and results in the lowest (or highest) average event probability overall. If the rankings are in conflict, then decision-makers could attempt to resolve this by considering making decisions for subgroups formed by the combinations of a small number of effect modifiers (whilst still adjusting for the full set of effect modifiers at the modelling stage). These chosen effect modifiers should be those that have the most impact on treatment effects in the population, based both on the strength of the interaction and on the range of covariate values in the population. The precise cut-points could be based on examining plots of the individual treatment effects against the effect modifiers in question one covariate at a time, holding the other covariates at the population means, or they could be guided by clinical reasoning or practice (say if there are established thresholds for normal vs. high values). Careful selection of subgroups in this manner is likely to be sufficient to resolve the conflict between decision questions, whilst keeping the resulting subgroup decisions simple enough for decision-makers to justify and implement.

**Figure 9:**
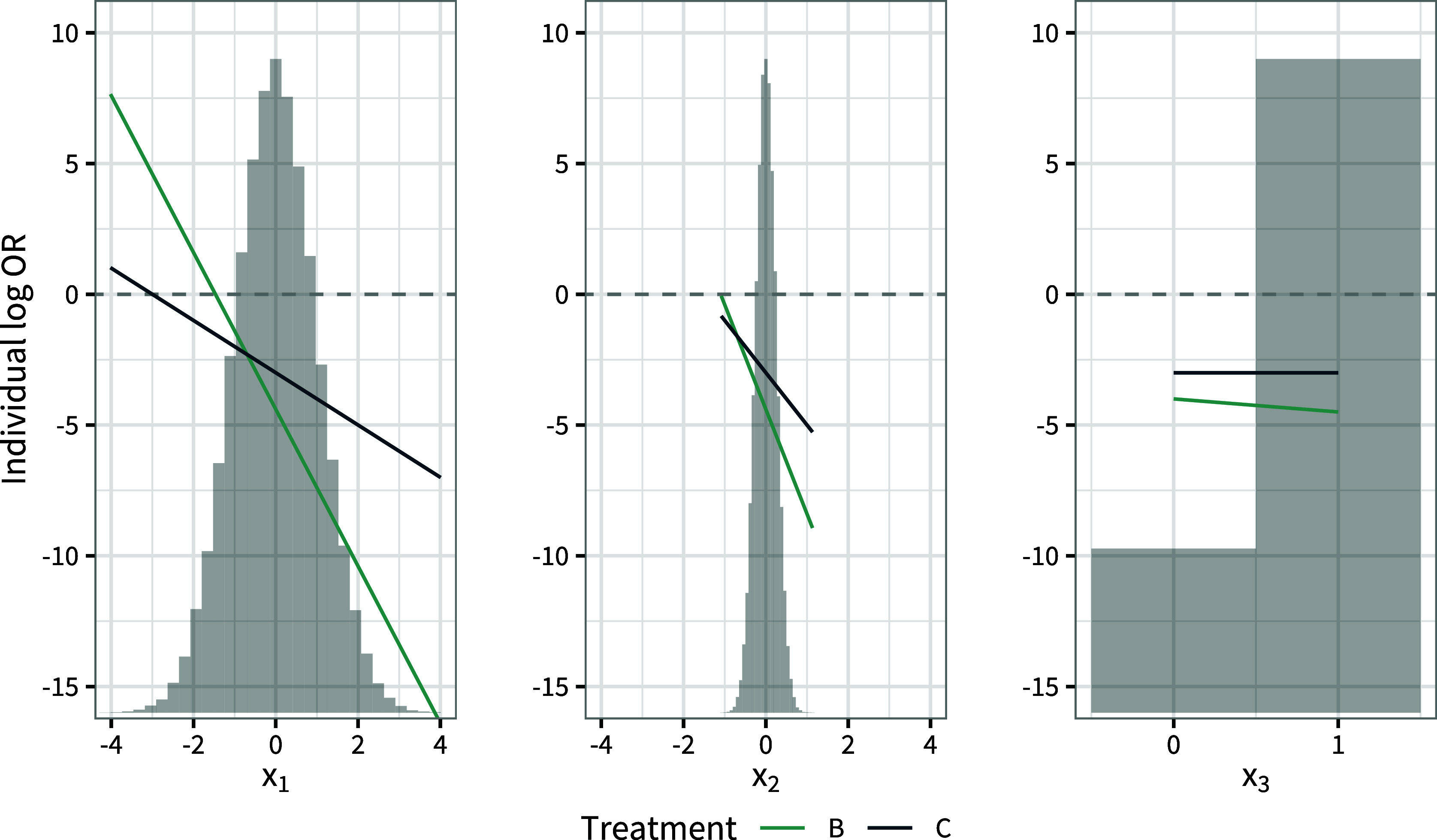
Example of considering a subgroup decision with three effect-modifying covariates. Each plot shows the individual-level treatment effects vs. treatment *A* (solid lines) over each of the covariate distributions in the population in turn (histograms), holding the other covariates at their population means.

For example, consider a scenario with two treatments *B* and *C* compared to reference *A*, and three potential effect modifiers which are distributed in the target population as 
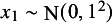
 , 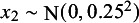 , 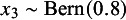 . There is a harmful binary outcome, modelled on the logit scale following model (1), with coefficients 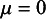 , 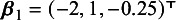 , 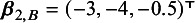 , 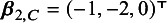 , 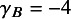 , and 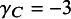 . The resulting population-average marginal and conditional log odds ratios are in conflict, 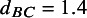 and 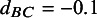 , so we proceed to consider a decision for subgroups. Plots of the individual-level treatment effects over each of the covariates in turn, holding the other covariates at their population means, are shown in Figure 9. Whilst 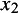 has the strongest interaction estimate, 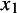 leads to greater variation in individual-level treatment effects over the population due to the greater variation in this covariate, and the individual-level treatment effects change ranks within the range of 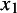 in the population. 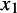 is therefore a good candidate for forming subgroups. The individual-level treatment effects cross at 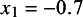 , and making separate decisions for subgroups at this cut-point (treatment *C* for 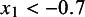 and *B* for 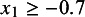 ) does resolve the conflict between population-average marginal and conditional effects ( 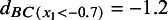 and 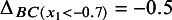 in the first subgroup, 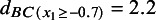 and 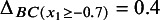 in the second). In many cases it may be more justifiable to base the precise cut-point(s) on clinical reasoning or practice. For example, in this case if 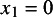 represents a clinically-meaningful threshold, forming subgroups around this cut-point also resolves the conflict ( 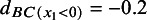 and 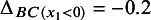 in the first subgroup, 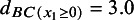 and 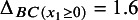 in the second).

When effect modification is not present, the two decision questions are aligned and both estimands will give the same ranking of treatments. In a Bayesian setting Bayesian *p*-values and the precision of the ranks will be identical between the population-average conditional and marginal effects, since the action of marginalisation is a monotonic transformation of the posterior about the origin.

### 7.2 Decisions based on cost-effectiveness

For decisions based on cost-effectiveness, patient heterogeneity (such as that caused by effect modification) should be handled appropriately by averaging net benefit over the population as in equation (8).^30^ Discrete event simulation based on individual or subgroup event probabilities or survival curves is one suitable approach,^31^ ^,^ ^32^ however such models can be complex to develop and evaluate. As a result, discrete event simulation is not as widely used as other approaches such as Markov models or decision trees, which typically do not account for patient heterogeneity and are based on average event probabilities 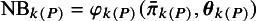 or average survival curves 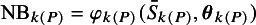 . Assuming that the structure of 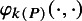 is maintained between approaches and that any additional parameters 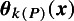 are averaged over the population in the same way, how different the resulting expected net benefit is depends on the non-linearity of 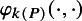 with respect to 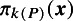 (due to Jensen’s inequality), with equality if 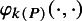 is linear in 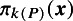 . However, there are likely to also be structural differences in 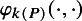 between approaches which may introduce further differences between the results.

Regardless of the cost-effectiveness model chosen, care must be taken to ensure that the relevant inputs are produced appropriately. In many cases these are the event probabilities on each treatment in the decision target population, either average 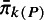 or individual 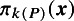 . These should be obtained by applying relevant relative treatment effect estimates (e.g., after population-adjustment into the target population) to a representative distribution for baseline risk. Evidence for this baseline risk distribution need not be obtained from the trial(s) used to estimate relative effects; indeed this may ideally be obtained from a representative registry or cohort study in the decision target population.^34^ Typically this baseline risk distribution is on the average event probability 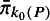 for a given treatment 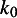 .

To then obtain average event probabilities on all other treatments, one simple approach is to rearrange equation (6) as 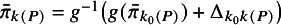 and apply population-average marginal treatment effect estimates to the baseline risk distribution. However, this is only correct if the population-average marginal effects 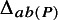 were produced by marginalising over the same baseline risk distribution, since these are not transportable across populations with different baseline risks (or covariate distributions). This means that population adjustment methods like MAIC and STC that can only estimate population-average marginal treatment effects in the aggregate study population of an indirect comparison are strictly limited to producing average event probabilities in this aggregate study population; the population-average marginal estimates are specific to this population, and cannot be applied to another population with a different distribution of baseline risk even if the covariate distributions are the same.

A more sophisticated approach when a regression model has been used is to instead apply equation (7), averaging absolute model predictions on each treatment over the target population.^5^ This requires that model (1) is estimated for all treatments, which is currently only possible using ML-NMR in a population adjustment setting. This also requires a distribution on the intercept 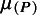 in the target population, instead of the baseline risk 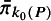 ; a procedure for converting between the two by solving equation (7) for values of 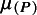 given a sample of values for 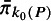 is given in Phillippo et al.^28^ Using this approach, average event probabilities can be produced in any target population of interest; an example using ML-NMR is given in Phillippo et al.^28^ In small networks like a two-study indirect comparison, ML-NMR may make use of an identifying assumption such as the shared effect modifier assumption to produce estimates for populations other than the aggregate study population (Section 3).

The same considerations apply for cost-effectiveness decisions involving survival outcomes. Furthermore, since the presence of covariate effects—the very motivation for performing population adjustment—implies that marginal hazard ratios are time-varying, the oft-used practice of applying a constant hazard ratio for each treatment to a baseline survival function in the economic model is not appropriate here. Instead, the estimated individual- or subgroup-specific survival curves 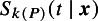 or population-average survival curves 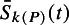 should be used directly in the modelling. MAIC and STC can obtain population-average survival curves on all treatments, but only in the population of the aggregate study. ML-NMR can estimate either individual-level or population-average survival curves on all treatments, in any population.^35^ As well as information on the covariate distribution in the target population, a distribution for the baseline hazard is required. When Kaplan-Meier data are available in the target population, even on a single treatment arm, this can be used to estimate the appropriate baseline hazard parameters within the ML-NMR model^35^; otherwise, baseline hazard estimates could be taken from a study in the network where the baseline hazard is deemed to be representative, whilst still allowing for differences in covariate distributions to be accounted for.

Carrying out a cost-effectiveness analysis does not resolve the fact that making a single treatment decision for an entire population may be sub-optimal, and that greater cost-effectiveness might be obtained by allowing different decisions for subgroups.

## 8 Discussion

In this article, we have argued that population-average conditional and marginal estimands correspond to different decision questions, either to maximise average effectiveness or to minimise (or maximise) average event probabilities respectively. We have demonstrated how the presence of effect modification means that these estimands and decision questions are no longer aligned, and may not correspond to the same ranking of treatments. Moreover, making a single treatment decision in the presence of effect modification can result in either selecting an inferior treatment for the majority of individuals, or selecting a treatment with a worse average event probability overall. Where allowable, making decisions by subgroups may result in patients being given a more effective treatment for them and result in greater cost-effectiveness overall. However, identification of valid subgroups is non-trivial; analyses to detect interactions and subgroups typically have low power, there is a risk of spurious findings particularly if many candidate factors are considered, and precision will be reduced within subgroups which may weaken conclusions.

Whether or not rank conflict between population-average conditional and marginal estimates actually occurs in a given setting depends on a range of factors (as illustrated in Section 4), including the strength of effect modification, the distribution of the effect modifiers, the distribution of baseline risk, and the strength and distribution of prognostic factors. Future research could include simulation studies across a range of realistic scenarios to give better insight into the likelihood of rank conflict occurring in practice, and the cases where this more likely to occur. We note however that, despite the wide range of contributing factors, rank conflict can only occur if the individual-level event probabilities or treatment effects change ranks for individuals within the population (Section 5); that is, there needs to be a sufficiently large proportion of the population for which the “best” treatment is different. This lends some practical intuition for when rank conflict may be expected to occur, and motivates our suggestion to use rank conflict as a simple diagnostic for when subgroup decisions may be desirable.

We have focused the motivation for this article on population-level decision making using population-adjusted analyses like MAIC or ML-NMR, which typically involve two or more trials and three or more treatments.^16^ However, our arguments apply equally to analyses of single trials, and where there are only two treatments (Section 4.3). Whilst trials do not typically consider adjusting for effect modifiers as a primary analysis, consideration of effect modifiers is central to generalising or transporting treatment effects to target populations (typically a marginal estimand is targeted through a propensity score analysis)^36^ ^,^ ^37^; such analyses are therefore subject to exactly the same issues that we describe here.

In the two-study indirect comparison scenario for which MAIC and STC are proposed, MAIC and STC (with simulation or G-computation) can produce estimates of population-average marginal treatment effects relevant to the aggregate study population. Whilst a targeted comparison against a competitor’s treatment in their study population may be desirable for commercial reasons, such analyses may not be relevant for decision-making where it is crucial that estimates are produced for a representative decision target population.^16^ However, MAIC and STC cannot produce estimates for another target population of interest, except in the special case of continuous outcomes and a linear model when the shared effect modifier assumption may be applied (Section 3). In the two-study indirect comparison scenario, ML-NMR can produce both conditional and marginal estimates relevant to the aggregate study population, and can produce estimates relevant to any target population of interest with the use of an additional identifying assumption such as the shared effect modifier assumption (Section 3). In larger networks, which may often be available in practice,^38^ ML-NMR may allow the shared effect modifier assumption to be assessed or avoided entirely.^28^ Larger networks where comparisons are informed by many trials may also allow the impact of effect modifiers to “balance out”; sparse networks or pairwise indirect comparisons with only one or two trials per comparison are potentially more vulnerable to bias resulting from effect modifier imbalance across studies.^16^ On the other hand, as more classes of treatments are included the potential set of effect modifiers grows larger, and without sufficient data additional shared effect modifier assumptions may be required within each class. We note that reliance on such identifying assumptions is only necessary due to the limitations of the available data, i.e., due to the lack of IPD sharing by manufacturers. IPD meta-regression is the ideal—but uncommon—special case of ML-NMR where IPD are available from every study, in which case there are sufficient data to estimate the model without additional identifying assumptions. To be relevant for decision-making, whichever method is used, estimates must be produced that are relevant to the decision target population.^16^

Previous discussion of non-collapsibility, such as the excellent paper by Daniel et al.,^13^ has largely focused on scenarios where there is no effect modification. In such cases, there are well-known results that i) conditional estimands lie further from the null than marginal estimands; ii) conditional estimators may have reduced precision compared to marginal estimators; iii) the reduction in precision is outweighed by the increased separation from the null, resulting in increased power for conditional estimands (as summarised by Daniel et al.^13^). Whilst comparing precision of population-average marginal and conditional estimators is not a meaningful comparison of like with like, this power comparison is meaningful because the two estimands share the same null. However, we have demonstrated that these results break down when there is effect modification, as the population-average marginal estimand is no longer always closer to the null—even if the distribution of effect modifiers is unchanged (Section 4.2).

Whilst for binary outcomes we focused on log odds ratios with a logit link function, the same issues and arguments apply to other non-collapsible effect measures such as the summary effect from a probit link model. The same arguments also apply to non-collapsible effect measures for other types of outcomes. For example, when analysing ordered categorical outcomes using ordered logistic or probit regression, there is a single summary population-average conditional treatment effect across all outcome categories, but the population-average marginal treatment effects differ for each category and the marginal rankings may change between categories.^28^ This should not be construed as a reason to prefer modelling collapsible effect measures such as risk differences or log risk ratios directly, e.g., by the use of an identity or log link function with a binary outome. Such models can result in predicted probabilities less than 0 or greater than 1, and will induce purely mathematical treatment-covariate interactions.

For survival or time-to-event outcomes analysed using (log) hazard ratios, we have seen that not only do population-average marginal hazard ratios depend on the shape of the hazard function and the distribution of baseline hazard and all prognostic and effect modifying covariates, but they must also vary over time. Crucially this means that, whenever covariates are present, proportional hazards *cannot* hold at the marginal level. Such covariates do not need to be effect modifying or time-varying; even with purely prognostic baseline covariates the mathematical consequence is that the population-average marginal hazard ratio is time-varying. A positive consequence of this, however, is that adjustment for covariates measured only at baseline can be sufficient to address violations of proportional hazards in unadjusted models (a phenomenon we have noted previously.^35^) Daniel et al.^13^ also considered the implications of non-collapsibility of the hazard ratio. They considered a marginal hazard ratio that had been additionally marginalised over time, as well as the covariates and baseline hazard, to provide a single non-time-varying marginal hazard ratio, and proposed an approach to obtain such a hazard ratio from an adjusted model. Daniel et al. note that such marginal hazard ratios (and thus any marginal rankings based on them) are further dependent on the length of study follow-up and observed censoring pattern, as well as the shape of the baseline hazard, and distributions of baseline risk and prognostic and effect modifying covariates.

For cost-effectiveness decisions, when effect modification or other sources of patient heterogeneity are present, the net benefit should be averaged over the population.^30^ This requires the production of individual- or subgroup-specific event probabilities; in the context of population-adjusted indirect comparisons or evidence syntheses of multiple studies, at present this is only possible using ML-NMR.^5^ Discrete event simulation^31^ ^,^ ^32^ is one suitable approach to constructing a net benefit function and averaging this over a population, however it is not widely used due to complexity. Markov models and decision trees are much more prevalent, however these approaches do not typically account for patient heterogeneity. Determining the possible extent of differences in the results between approaches, and when they might be used interchangeably, is an interesting area for further research. Regardless of the type of economic model used, or indeed whether there is effect modification at all, the relevant effectiveness inputs must be produced appropriately. MAIC and STC cannot produce estimates of individual event probabilities, and are limited to producing average event probabilities in the aggregate study population of an indirect comparison. The population-average marginal treatment effects that these methods estimate are specific to the distribution of baseline risk (as well as all covariates) in the aggregate study population, and cannot be applied to a different baseline risk distribution in a target population even if the covariates are similar. At present, ML-NMR is the only population adjustment method that can produce individual or average event probabilities, and can do so in any target population of interest.^5^ ^,^ ^28^

For decisions based purely on effectiveness, when effect modification is present we propose that decision-makers look at both the population-average conditional and marginal estimates and their respective treatment rankings to assess whether these are in conflict (Section 7). If the rankings agree, then there is no substantial conflict and a single treatment decision may be justified for the entire population. However, if the rankings are in conflict then a single treatment decision cannot satisfy both decision questions for the entire population, and decision-makers may wish to consider splitting decisions into subgroups. This proposal necessitates using an analysis method that can produce both conditional and marginal estimates. For analyses of single trials, this is possible using standard regression adjustment, followed by the marginalisation approach of Zhang^39^ (for binary outcomes) or Daniel et al.^13^ (for time-to-event outcomes). For population-adjusted indirect comparisons or evidence syntheses of multiple studies, current implementations of MAIC and STC cannot produce estimates of both estimands, and moreover cannot typically produce estimates for a chosen decision target population. ML-NMR can produce estimates of both the conditional and marginal estimands in any target population of interest, as well as the necessary quantities for cost-effectiveness models such as average event probabilities or subgroup/individual event probabilities, making this a powerful tool for analysts and decision-makers.^5^ ^,^ ^28^

## Data Availability

Data availability is not applicable to this article as no new data were created or analysed in this study.

## Appendix

### A Definitions of estimands in terms of potential outcomes

We can define each of the estimands given in Section 2 using the potential outcomes framework.^40^ Let  denote potential outcomes for individuals receiving treatment *k*, and let  be a suitable link function such as the logit link function.

The individual-level conditional treatment effects (3) for an individual with covariates  receiving treatment *b* compared to *a* are defined as

(A1)

where the expectations are over the distribution of potential outcomes for an individual conditional on the covariates.

The population-average conditional treatment effects (4) for treatment *b* compared to *a* in population *P* are defined as

(A2)

where the inner expectations are over the distribution of potential outcomes for an individual conditional on the covariates, and the outer expectations are over the population *P*. In other words, this is the expectation of the individual-level conditional treatment effects over the population,  .

The population-average marginal treatment effects (6) for treatment *b* compared to *a* in population *P* are defined as

(A3)

where the expectations are over the distribution of potential outcomes in the population *P*.

### B Proof that effect modification is necessary for rank-switching

We prove here that rank-switching between the population-average conditional and marginal effects can only occur in the presence of effect modification, between treatments that have different interaction terms (i.e., no shared effect modifier assumption), and when this causes treatment ranks to change across individuals/subgroups in the population. We shall consider the population-average conditional and marginal estimands  and  for any two treatments *a* and *b*, and consider without loss of generality that we have  .

Firstly, let us show that when there is no effect modification between treatments *a* and *b*, there can be no rank switching between the population-average conditional and marginal estimands for these two treatments (i.e.,  and  must have the same sign). Let  so that there is no effect modification between these two treatments. (Either there is no effect modification at all so  , or the shared effect modifier assumption is made for these two treatments so  .) In this case, we have that

(B.1)

following the definition of  in equation (4). We have  , so therefore

(B.2)

Since  is monotonically increasing by definition as a link function, and following the definition of the individual event probabilities in equation (5), this means that

(B.3)

and therefore

(B.4)

following the definition of the average event probabilities in equation (7). From the definition of  in (6), and again since  is monotonically increasing, this means that

(B.5)

Therefore, if there is no effect modification then  and  cannot be in conflict; they must have the same sign and the corresponding treatment ranks must be in agreement for these two treatments. A similar argument extends this fact to the case where  is non-zero but  is constant within the population, so that there is no substantive effect modification within the population.

Secondly, let us show that effect modification is necessary for the two estimands  and  to be in conflict (i.e., to have opposite signs), and that this effect modification must cause the individual-level treatment effects to change ranks across individuals/subgroups within the population for the conflict to occur. Consider still that  , but now that  . From the fact that  is monotonically increasing,  means that

(B.6)

following definition (7). Therefore, there must be some values of  within the population, say the set  , where

(B.7)

and therefore where

(B.8)

again using the fact that  is monotonically increasing. We also must have that the set  does not constitute the entire population, because if this was the case then equation (B.8) implies that  (whereas we have assumed the contrary). Together we have  for  in  and  for  not in  , in other words the individual treatment ranks change for different individuals/subgroups in the population, and this necessarily means that  is not constant. By definition (equation (3)), this requires that  is non-zero, and that  is not constant in the population; that is, there is substantive effect modification between the two treatments in the population.

We have therefore shown that effect modification is a necessary condition for rank-switching between the population-average conditional and marginal effects to occur, and moreover the effect modification must give rise to different treatment rankings for different individuals/subgroups in the population if the rank-switching is to occur.

## References

[1] Bucher HC , Guyatt GH , Griffith LE , Walter SD . The results of direct and indirect treatment comparisons in meta-analysis of randomized controlled trials. J. Clin. Epidemiol. 1997;50(6):683–691. 10.1016/s0895-4356(97)00049-8. 9250266

[2] Higgins JPT , Whitehead A . Borrowing strength from external trials in a meta-analysis. Stat. Med. 1996;15(24):2733–2749. 10.1002/(sici)1097-0258(19961230)15:24<2733::aid-sim562>3.0.co;2-0. 8981683

[3] Lu GB , Ades AE . Combination of direct and indirect evidence in mixed treatment comparisons. Stat. Med. 2004;23(20):3105–3124. 10.1002/sim.1875. 15449338

[4] Dias S , Welton NJ , Sutton AJ , Ades AE . Technical Support Document 2: A Generalised Linear Modelling Framework for Pair-Wise and Network Meta-Analysis of Randomised Controlled Trials. Technical report. NICE Decision Support Unit, Sheffield; 2011. 27466657

[5] Phillippo DM , Dias S , Ades AE , et al. Multilevel network meta-regression for population-adjusted treatment comparisons. J. Royal Stat. Soc. Ser. A (Stat. Soc.) 2020;183(3):1189–1210. 10.1111/rssa.12579. PMC7362893 32684669

[6] Signorovitch JE , Wu EQ , Yu AP , et al. Comparative effectiveness without head-to-head trials a method for matching-adjusted indirect comparisons applied to psoriasis treatment with adalimumab or etanercept. Pharmacoeconomics 2010;28(10):935–945. 10.2165/11538370-000000000-00000. 20831302

[7] Caro JJ , Ishak KJ . No head-to-head trial? Simulate the missing arms. Pharmacoeconomics 2010;28(10):957–967. 20831304 10.2165/11537420-000000000-00000

[8] Ishak KJ , Proskorovsky I , Benedict A . Simulation and matching-based approaches for indirect comparison of treatments. Pharmacoeconomics 2015;33(6):537–549. 10.1007/s40273-015-0271-1. 25795232

[9] Remiro-Azócar A , Heath A , Baio G . Parametric G-computation for compatible indirect treatment comparisons with limited individual patient data. Res. Synth. Methods 2022;13(6):716–744. 10.1002/jrsm.1565. 35485582 PMC9790405

[10] Gail MH , Wieand S , Piantadosi S . Biased estimates of treatment effect in randomized experiments with nonlinear regressions and omitted covariates. Biometrika 1984;71(3):431–444. 10.1093/biomet/71.3.431.

[11] Greenland S , Pearl J , Robins JM . Confounding and collapsibility in causal inference. Stat. Sci. 1999;14(1):29–46. 10.1214/ss/1009211805.

[12] Neuhaus JM , Jewell NP . A geometric approach to assess bias due to omitted covariates in generalized linear models. Biometrika 1993;80(4):807–815. 10.1093/biomet/80.4.807.

[13] Daniel R , Zhang J , Farewell D . Making apples from oranges: Comparing noncollapsible effect estimators and their standard errors after adjustment for different covariate sets. Biom. J. 2020;63(3):528–557. 10.1002/bimj.201900297. 33314251 PMC7986756

[14] Phillippo DM , Dias S , Ades AE , Welton NJ . Target estimands for efficient decision making: Response to comments on “assessing the performance of population adjustment methods for anchored indirect comparisons: A simulation study”. Stat. Med. 2021;40(11):2759–2763. 10.1002/sim.8965. 33963586 PMC9495275

[15] Remiro-Azócar A , Heath A , Baio G . Conflating marginal and conditional treatment effects: Comments on “assessing the performance of population adjustment methods for anchored indirect comparisons: A simulation study”. Stat. Med. 2021;40(11):2753–2758. 10.1002/sim.8857. 33963582

[16] Phillippo DM , Ades AE , Dias S , Palmer S , Abrams KR , Welton NJ . Technical Support Document 18: Methods for Population-Adjusted Indirect Comparisons in Submission to NICE. Technical report. NICE Decision Support Unit, Sheffield; 2016.

[17] Berlin JA , Santanna J , Schmid CH , Szczech LA , Feldman HI . Individual patient- versus group-level data meta-regressions for the investigation of treatment effect modifiers: ecological bias rears its ugly head. Stat. Med. 2002;21(3):371–387. 10.1002/sim.1023. 11813224

[18] Lambert PC , Sutton AJ , Abrams KR , Jones DR . A comparison of summary patient-level covariates in meta-regression with individual patient data meta-analysis. J. Clin. Epidemiol. 2002;55(1):86–94. 10.1016/S0895-4356(01)00414-0. 11781126

[19] Riley RD , Lambert PC , Abo-Zaid G . Meta-analysis of individual participant data: rationale, conduct, and reporting. Brit. Med. J. 2010;340:c221. 10.1136/bmj.c221. 20139215

[20] Dias S , Sutton AJ , Welton NJ , Ades AE . Technical Support Document 3: Heterogeneity: Subgroups, Meta-Regression, Bias and Bias-Adjustment. Technical report. NICE Decision Support Unit, Sheffield; 2011.

[21] Phillippo DM , Ades AE , Dias S , Palmer S , Abrams KR , Welton NJ . Methods for population-adjusted indirect comparisons in health technology appraisal. Med. Decis.Making 2018;38(2):200–211. 10.1177/0272989x17725740. 28823204 PMC5774635

[22] Sutton AJ , Kendrick D , Coupland CAC . Meta-analysis of individual- and aggregate-level data. Stat. Med. 2008;27(5):651–669. 10.1002/sim.2916. 17514698

[23] Saramago P , Sutton AJ , Cooper NJ , Manca A . Mixed treatment comparisons using aggregate and individual participant level data. Stat. Med. 2012;31(28):3516–3536. 10.1002/sim.5442. 22764016

[24] Donegan S , Williamson P , D’Alessandro U , Garner P , Tudur SC . Combining individual patient data and aggregate data in mixed treatment comparison meta-analysis: Individual patient data may be beneficial if only for a subset of trials. Stat. Med. 2013;32(6):914–930. 10.1002/sim.5584. 22987606

[25] Phillippo DM . multinma: Network meta-analysis of individual and aggregate data in stan. R Package. 2020. Available from https://cran.r-project.org/package=multinma. 10.5281/zenodo.3904454.

[26] Zhang L , Bujkiewicz S , Jackson D . Four alternative methodologies for simulated treatment comparison: How could the use of simulation be re-invigorated? Res. Synth. Methods 2024;15(2):227–241. 10.1002/jrsm.1681. 38104969

[27] Ren S , Ren S , Welton NJ , Strong M . Advancing unanchored simulated treatment comparisons: A novel implementation and simulation study. Res. Synth. Methods 2024;15(4):657–670. 10.1002/jrsm.1718. 38590103

[28] Phillippo DM , Dias S , Ades AE , et al. Validating the assumptions of population adjustment: application of multilevel network meta-regression to a network of treatments for plaque psoriasis. Med. Decis. Making 2023;43(1):53–67. 10.1177/0272989X221117162. 35997006 PMC9742635

[29] Harari O , Soltanifar M , Cappelleri JC , et al. Network meta-interpolation: Effect modification adjustment in network meta-analysis using subgroup analyses. Res. Synth. Methods 2023;14(2):211–233. 10.1002/jrsm.1608. 36283960

[30] Welton NJ , Soares MO , Palmer S , et al. Accounting for heterogeneity in relative treatment effects for use in cost-effectiveness models and value-of-information analyses. Med. Decis. Making 2015;35(5):608–621. 10.1177/0272989x15570113. 25712447 PMC4471065

[31] Karnon J , Stahl J , Brennan A , Caro JJ , Mar J , Möller J . Modeling using discrete event simulation: A report of the ISPOR-SMDM modeling good research practices task force-4. Value Health 2012;15(6):821–827. 10.1016/j.jval.2012.04.013. 22999131

[32] Karnon J , Afzali HHA . When to use discrete event simulation (DES) for the economic evaluation of health technologies? A review and critique of the costs and benefits of DES. Pharmacoeconomics 2014;32(6):547–558. 10.1007/s40273-014-0147-9, 24627341

[33] National Institute for Health and Care Excellence. NICE Health Technology Evaluations: The Manual. Technical report. National Institute for Health and Care Excellence, London; 2022.

[34] Dias S , Welton NJ , Sutton AJ , Ades AE . Technical Support Document 5: Evidence Synthesis in the Baseline Natural History Model. Technical report. NICE Decision Support Unit, Sheffield; 2011.

[35] Phillippo DM , Dias S , Ades AE , Welton NJ . Multilevel network meta-regression for general likelihoods: synthesis of individual and aggregate data with applications to survival analysis. 2024. 10.48550/ARXIV.2401.12640.

[36] Cole SR , Stuart EA . Generalizing evidence from randomized clinical trials to target populations. Amer. J. Epidemiol. 2010;172(1):107–115. 10.1093/aje/kwq084. 20547574 PMC2915476

[37] Stuart EA , Cole SR , Bradshaw CP , Leaf PJ . The use of propensity scores to assess the generalizability of results from randomized trials. J. Royal Stat. Soc. Ser. A—Stat. Soc. 2011;174:369–386. 10.1111/j.1467-985X.2010.00673.x. PMC4051511 24926156

[38] Phillippo DM , Dias S , Elsada A , Ades AE , Welton NJ . Population adjustment methods for indirect comparisons: A review of national institute for health and care excellence technology appraisals. Int. J. Technol. Assess. Health Care 2019;35(3):221–228. 10.1017/S0266462319000333 31190671 PMC6650293

[39] Zhang Z. Estimating a marginal causal odds ratio subject to confounding. Commun. Stat. Theory Methods 2008;38(3):309–321. 10.1080/03610920802200076.

[40] Rubin DB . Estimating causal effects of treatments in randomized and nonrandomized studies. J. Educ. Psychol. 1974;66(5):688–701. 10.1037/h0037350.

